# Supplementary material for: A mechanism with severing near barbed ends and annealing explains structure and dynamics of dendritic actin networks
Source: eLife. 2022 Jun 7;11:e69031. doi: 10.7554/eLife.69031 (PMC9252579; doi:10.7554/eLife.69031)
Supplement: Transparent reporting form [file elife-69031-transrepform1.docx]

***eLife’s* transparent reporting form**

We encourage authors to provide detailed information *within their submission* to facilitate the interpretation and replication of experiments. Authors can upload supporting documentation to indicate the use of appropriate reporting guidelines for health-related research (see [EQUATOR Network](about:blank)), life science research (see the BioSharing Information Resource), or the [ARRIVE guidelines](about:blank) for reporting work involving animal research. Where applicable, authors should refer to any relevant reporting standards documents in this form.

If you have any questions, please consult our Journal Policies and/or contact us: [editorial@elifesciences.org](about:blank).

**Sample-size estimation**

- You should state whether an appropriate sample size was computed when the study was being designed
- You should state the statistical method of sample size computation and any required assumptions
- If no explicit power analysis was used, you should describe how you decided what sample (replicate) size (number) to use

Please outline where this information can be found within the submission (e.g., sections or figure legends), or explain why this information doesn’t apply to your submission:

We have listed the sample size information in the figure legends and method section.

In simulations we used 5 independent runs in steady state for each parameter set. This number was sufficient to smooth out stochastic fluctuations such that figure panels show a clear visual trend of plotted quantities as function of distance and time.

For the experimental data reported in Figure 6A, a sample size of 124 SiMS was

determined to be sufficiently large, by measuring a number of disassembled speckles within 1 s that was larger than the estimated disassembly of 3.72 speckles within 1 s (95% CI, 0 - 7.6 speckles), based on overall disassembly rate of 0.03 /s from Watanabe and Mitchison, Science 295:1083 2002.

The sample size for the number of actin SiMS in Figure 6C was determined by using GraphPad StatMate and published data by Kueh et al. (J. Cell Biol. 182: 341–353, 2008). In the data by Kueh et al, we estimate the decay of actin photoactivation of fluorescence signals was slowed >2-fold by CD, which roughly corresponds to >0.7 survival in CD-treated cells at the time of control half-life. We therefore acquired timelapse data where we can measure at least 40 actin SiMS in each situation. Comparison was further made by t-test of 3 independent cell data. For Figure 6-supplement 1C, we also acquired at least 40 speckles in each situation to resolve a several-fold larger difference in survival rate

**Replicates**

- You should report how often each experiment was performed
- You should include a definition of biological versus technical replication
- The data obtained should be provided and sufficient information should be provided to indicate the number of independent biological and/or technical replicates
- If you encountered any outliers, you should describe how these were handled
- Criteria for exclusion/inclusion of data should be clearly stated
- High-throughput sequence data should be uploaded before submission, with a private link for reviewers provided (these are available from both GEO and ArrayExpress)

Please outline where this information can be found within the submission (e.g., sections or figure legends), or explain why this information doesn’t apply to your submission:

We have reported this information in the figure legends and methods section. Simulation results are from data collected from 5 independent runs in steady state for each parameter set. The simulation was considered in steady state once the simulation time exceeded the time for retrograde flow to move a filament from the leading edge to twice the expected lamellipodial length.

**Statistical reporting**

- Statistical analysis methods should be described and justified
- Raw data should be presented in figures whenever informative to do so (typically when N per group is less than 10)
- For each experiment, you should identify the statistical tests used, exact values of N, definitions of center, methods of multiple test correction, and dispersion and precision measures (e.g., mean, median, SD, SEM, confidence intervals; and, for the major substantive results, a measure of effect size (e.g., Pearson's r, Cohen's d)
- Report exact p-values wherever possible alongside the summary statistics and 95% confidence intervals. These should be reported for all key questions and not only when the p-value is less than 0.05.

Please outline where this information can be found within the submission (e.g., sections or figure legends), or explain why this information doesn’t apply to your submission:

We have reported this information in the figure legends and methods section.

(For large datasets, or papers with a very large number of statistical tests, you may upload a single table file with tests, Ns, etc., with reference to sections in the manuscript.)

**Group allocation**

- Indicate how samples were allocated into experimental groups (in the case of clinical studies, please specify allocation to treatment method); if randomization was used, please also state if restricted randomization was applied
- Indicate if masking was used during group allocation, data collection and/or data analysis

Please outline where this information can be found within the submission (e.g., sections or figure legends), or explain why this information doesn’t apply to your submission:

Not applicable.

**Additional data files (“source data”)**

- We encourage you to upload relevant additional data files, such as numerical data that are represented as a graph in a figure, or as a summary table
- Where provided, these should be in the most useful format, and they can be uploaded as “Source data” files linked to a main figure or table
- Include model definition files including the full list of parameters used
- Include code used for data analysis (e.g., R, MatLab)
- Avoid stating that data files are “available upon request”

Please indicate the figures or tables for which source data files have been provided:

We provide the Java code to reproduce all the simulation data in the main and supplemental figures at https://github.com/vavylonis/LamellipodiumSeverAnneal. The full list of parameters is included in the file *FilParam.txt* with parameters specific to the Keratocyte and XTC parameter sets listed in Table 1 of the main text. The function of each java class file in the folder is described in the *ReadMe.txt* file.
